# Prediction of early response to overall treatment for schizophrenia: A functional magnetic resonance imaging study

**DOI:** 10.1002/brb3.1211

**Published:** 2019-01-30

**Authors:** Long‐Biao Cui, Min Cai, Xing‐Rui Wang, Yuan‐Qiang Zhu, Liu‐Xian Wang, Yi‐Bin Xi, Hua‐Ning Wang, Xia Zhu, Hong Yin

**Affiliations:** ^1^ Department of Radiology, Xijing Hospital Fourth Military Medical University Xi’an China; ^2^ School of Medical Psychology Fourth Military Medical University Xi’an China; ^3^ Department of Psychiatry Xijing Hospital, Fourth Military Medical University Xi’an China

**Keywords:** ALFF, fMRI, prediction, response, schizophrenia, treatment

## Abstract

**Introduction:**

Treatment response at an early stage of schizophrenia is of considerable value with regard to future management of the disorder; however, there are currently no biomarkers that can inform physicians about the likelihood of response.

**Objects:**

We aim to develop and validate regional brain activity derived from functional magnetic resonance imaging (fMRI) as a potential signature to predict early treatment response in schizophrenia.

**Methods:**

Amplitude of low‐frequency fluctuation (ALFF) was measured at the start of the first/single episode resulting in hospitalization. Inpatients were included in a principal dataset (*n* = 79) and a replication dataset (*n* = 44). Two groups of healthy controls (*n* = 87; *n* = 106) were also recruited for each dataset. The clinical response was assessed at discharge from the hospital. The predictive capacity of normalized ALFF in patients by healthy controls, ALFF_ratio_, was evaluated based on diagnostic tests and clinical correlates.

**Results:**

In the principal dataset, responders exhibited increased baseline ALFF in the left postcentral gyrus/inferior parietal lobule relative to non‐responders. ALFF_ratio_ of responders before treatment was significantly higher than that of non‐responders (*p* < 0.001). The area under the receiver operating characteristic curve was 0.746 for baseline ALFF_ratio_ to distinguish responders from non‐responders, and the sensitivity, specificity, and accuracy were 72.7%, 68.6%, and 70.9%, respectively. Similar results were found in the independent replication dataset.

**Conclusions:**

Baseline regional activity of the brain seems to be predictive of early response to treatment for schizophrenia. This study shows that psycho‐neuroimaging holds promise for influencing the clinical treatment and management of schizophrenia.

## 1 INTRODUCTION

Despite great progress in the understanding of the pathophysiology of schizophrenia, optimization of treatment for this disorder still remains a challenge (Owen, Sawa, & Mortensen, 2016). Quantifying the relationship between brain changes and different treatment responses, and understanding whether these changes can be used as predictive biomarkers for treatment response could help to address this challenge. Predictive biomarkers are widely used to influence the treatment selection for a variety of diseases, namely cancer, but even in the assessment of experimental thermal pain (Wager et al., 2013). Dazzan *et al.* concluded that alterations of gray and white matter identified on structural neuroimaging, predominantly distributed in the medial temporal and prefrontal cortices, are potential neuroanatomical markers for the prediction of poor symptomatic and functional outcome (Dazzan et al., 2015). There have also been studies that identified correlations between structural features and antipsychotic treatment response from the perspectives of both volume and morphology, involving asymmetry and hypogyria (Altamura et al., 2017; Dusi et al., 2017; Francis et al., 2018; Fung et al., 2014; Hutcheson, Clark, Bolding, White, & Lahti, 2014; Molina, Taboada, Aragues, Hernandez, & Sanz‐Fuentenebro, 2014; Morch‐Johnsen et al., 2015; Palaniyappan et al., 2013; Premkumar et al., 2015). However, functional brain imaging‐based prognostic markers require further investigation before being used clinically for individualized psychosis treatment decisions (Keefe & Kahn, 2017).

Functional magnetic resonance imaging (fMRI) has shown to be a promising technique to demonstrate neuronal activity and also to identify the therapeutic response in psychosis patients. Recent studies have investigated the use of striatal functional connectivity and blood oxygen level‐dependent (BOLD) activation in the anterior cingulate cortex as predictors of identifying patients suffering from acute psychosis who will respond to antipsychotics (Sarpal et al., 2016; Shafritz et al., 2018). The changes in symptoms of patients with schizophrenia or schizoaffective disorder are correlated with the ventral tegmental area/midbrain connectivity with dorsal anterior cingulate cortex (Hadley et al., 2014), as well as hippocampal connectivity (Kraguljac, White, Hadley, Hadley et al., 2016). Another study examining resting‐state networks suggests that negative symptom improvement in schizophrenia patients after seven months of treatment could be accurately classified by bilateral frontoparietal and default mode networks (Nejad et al., 2013). In a classification study, it has been shown that the lower classification scores of intrinsic connectivity networks predicted the better treatment outcome in schizophrenia patients (Li, Jing et al., 2017). Additionally, the dorsal attention network played an important role in reflecting symptoms changes in schizophrenia (Kraguljac, White, Hadley, Visscher et al., 2016). Most recently, links between connectome organization of resting‐state networks and predicting short‐term clinical outcomes of schizophrenia have been established (Doucet, Moser, Luber, Leibu, & Frangou, 2018). All of these studies focusing on the clinical implications of connectivity or large‐scale networks in schizophrenia indicate that fMRI‐based brain changes are a pivotal signature for predicting antipsychotic treatment response.

However, the relationship between regional activity within brain function, that is amplitude of low‐frequency fluctuation (ALFF) directly reflecting local field spontaneous neural activity (Logothetis, Pauls, Augath, Trinath, & Oeltermann, 2001), and its potential value for prediction has not yet been established. ALFF mathematically measures the strength of low‐frequency oscillations with a high degree of synchrony of the fMRI time series, which is higher in gray matter than in white matter (Biswal, Yetkin, Haughton, & Hyde, 1995). The neural activation in the visual cortex has been found to be related to ALFF at around 0.034 Hz (Kiviniemi et al., 2000), implying that ALFF represents regional spontaneous neuronal activity. On the basis of neuro‐electrophysiological findings, ALFF is considered to be biologically or physiologically significant in healthy brain (Mohamed, Yousem, Tekes, Browner, & Calhoun, 2004) and also involved in the pathogenesis of schizophrenia as reported by an increasing number of recent studies (Alonso‐Solis et al., 2017; Chyzhyk, Grana, Ongur, & Shinn, 2015; Fu et al., 2018; Li, Lei et al., 2017; Lian et al., 2018; Lui et al., 2015; Sui et al., 2018; Yu et al., 2014; Zhang et al., 2018). Previous studies have observed a marked attenuation of ALFF in the postcentral gyrus and parahippocampus and an augmentation in the putamen in medication‐naïve first‐episode schizophrenia patients relative to a healthy population (Cui et al., 2016), as well as abnormal ALFF within resting‐state networks (Cui, Liu et al., 2017). A 1‐year longitudinal study investigated the altered ALFF in the inferior parietal lobule, orbitofrontal cortex, and inferior occipital gyrus in first‐episode schizophrenia patients (Li et al., 2016). These studies indicate that ALFF could be a potential feature associated with treatment response for schizophrenia, but further investigation is still required.

To this end, an important step is to perform a clinical assessment of the patient upon the first/single hospitalization to obtain information about their early response to treatment for schizophrenia. This will help to predict the future outcome. Considering schizophrenia as a debilitating disorder, the time window of opportunity for course‐altering intervention is crucial for the outcome of this disease (Millan et al., 2016). An alternative approach is to try to predict the treatment response as early as possible. However, for the studies mentioned above which include data from 6 weeks (Hadley et al., 2014; Kraguljac, White, Hadley, Visscher et al., 2016; Kraguljac, White, Hadley, Hadley et al., 2016; Li, Jing et al., 2017), 12 weeks (Sarpal et al., 2016), and 7 months (Nejad et al., 2013), the response prediction at an early stage in schizophrenia remains unknown.

Therefore, in this study, we firstly aimed to examine whether ALFF could serve as a predictor for early treatment response in a principal dataset of patients. Our second aim was to test the results in an independent second patient cohort, the replication dataset, to validate the generalizability. We hypothesized that patients with a distinct feature of regional brain activity, that is ALFF, would reveal different responsiveness to treatment for the current hospital admission.

## 2 MATERIAL AND METHODS

### 2.1 Pipeline

**Figure 1:**
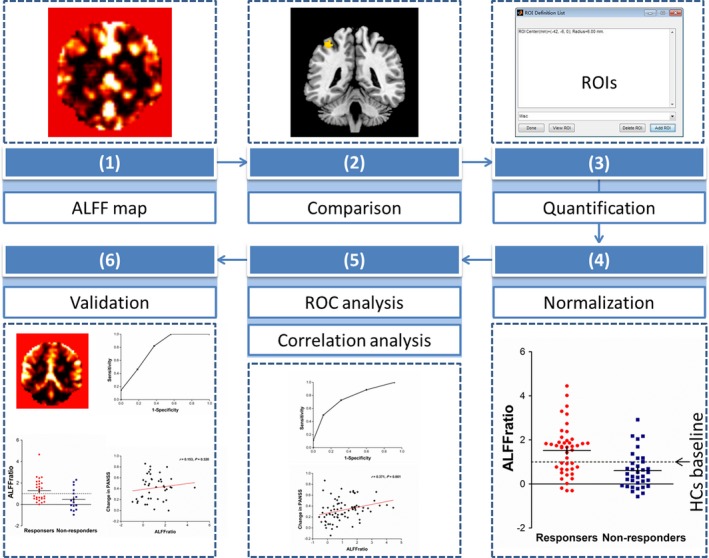
Overview of data analysis. (1) Amplitude of low‐frequency fluctuation map for each subject. (2) Identification of markers by comparison between responders and non‐responders. (3) Quantification of potential markers by extracting values of regions of interest (ROIs) made from peak difference. (4) Normalization of markers weighted by healthy controls (HCs). (5) Evaluation of predictor based on ROC and correlation analyses. (6) Testing in independent replication sample using the marker identified

The following steps outline how we developed and validated the potential brain marker in Figure 1. (a) In the principal dataset, the whole brain ALFF was calculated, yielding an ALFF map for each participant. (b) A voxel‐based comparison of ALFF was performed between responders and non‐responders to obtain targets of ALFF, which might be used for predicting response. (c) ALFF values of all the subjects were extracted using the regions of interest (ROIs) that were made of peak coordinates in clusters with ALFF differences between responders and non‐responders outlined in step (b). (d) ALFF values in each cluster of patients were normalized to the mean ALFF value of the healthy controls, that is, the ratio of ALFF, ALFF_ratio_ = ALFF_patients_ ÷ ALFF_healthy controls_. (e) Receiver operating characteristic (ROC) analysis was performed and correlation between ALFF_ratio_ and changes in clinical scales, length of hospital stay, and antipsychotic dosage were analyzed. (f) In the replication dataset, we tested the clinical generalizability of ALFF_ratio_ in the key region with the highest level of diagnostic performance and clinical correlation identified in step (e).

### 2.2 Participants

This study was approved by the Institutional Ethics Committee, First Affiliated Hospital (Xijing Hospital) of the Fourth Military Medical University. Written informed consent was obtained from all the participants (or their parents for those under age of 18 years) after complete description of the study. Between April 2011 and December 2013, 100 patients with schizophrenia were recruited from the Department of Psychiatry at Xijing Hospital. Ninety‐two healthy controls recruited from the local community also participated in this study. The participants were diagnosed on the basis of Structured Clinical Interview for Diagnostic and Statistical Manual of Mental Disorders, Fourth Edition, Text Revision (DSM‐IV‐TR), and consensus diagnoses were made by two experienced clinical psychiatrists using all the available information. Diagnosis of schizophrenia for patients with illness duration of <6 months was confirmed by follow‐up clinical assessment. All patients underwent MRI scanning at baseline. Afterward, our study had no influence on the therapy and clinical assessment determined by their clinicians according to standard clinical practice.

It has been suggested that dopamine D2 receptor occupancy between 60% and 70% is associated with optimal subjective experience (de Haan et al., 2004, 2003). APA Practice Guidelines proposes that olanzapine is an effective antipsychotic when administered in doses of 10–20 mg/day in the acute phase of schizophrenia (Lehman et al., 2004), and there is small difference in the effectiveness of individual antipsychotics (Leucht et al., 2013). Table 1 shows that the majority of patients received second‐generation antipsychotics in doses recommended by APA Practice Guidelines (olanzapine equivalents, 9.4–11.8 mg/d) (Leucht, Samara, Heres, & Davis, 2016), in line with a recent study (Doucet et al., 2018). Positive And Negative Syndrome Scale (PANSS) and Hamilton Depression Rating Scale (HAMD)/Hamilton Anxiety Rating Scale (HAMA) (needed by partial patients) that evaluated symptoms of patients at the time of MRI scans and before discharging were used for the following analysis. Percentage change on PANSS score was used to assess treatment responses: PANSS percentage change = (PANSS endpoint score — PANSS baseline score) × 100 ÷ (PANSS baseline score — 30) (Obermeier et al., 2010; Yu et al., 2018). A responder was defined if there was >30% reduction in PANSS total scores (Beitinger, Lin, Kissling, & Leucht, 2008; Leucht, Arbter, Engel, Kissling, & Davis, 2009). The exclusion criteria included the following: (a) presence of another axis I or axis II psychiatric disorder; (b) history of receiving electroconvulsive therapy (ECT); (c) history of clinically significant neurological, neurosurgical, or medical illnesses; (d) substance abuse within the prior 30 days or substance dependence within the prior 6 months; (e) pregnancy or MR imaging contraindications, for example, cardiac pacemakers and other metallic implants; (f) unwillingness to undertake the scanning. Excluding help seeking in 3 outpatients, incomplete clinical data in 14 patients, and excessive head motion in four patients and five healthy controls resulted in 79 patients and 87 healthy controls in this study. Demographic and clinical characteristics are listed in Table 1.

**Table 1:** Demographical and clinical data of participants

Characteristic	Principal dataset				Replication dataset			
	Responders (*n* = 44)	Non‐responders (*n* = 35)	*p* values	HCs (*n* = 87)	Responders (*n* = 28)	Non‐responders (*n* = 16)	*p* values	HCs (*n* = 106)
Age (y)	25.2 ± 5.9	26.0 ± 7.0	0.559	28.8 ± 6.6	21.9 ± 5.7	26.7 ± 9.6	0.014	29.6 ± 10.5
Gender (M/F)	24/20	19/16	0.982	51/36	19/9	9/7	0.441	54/52
Education level (y)	13.3 ± 1.9	12.9 ± 1.9	0.488	13.1 ± 3.5	12.0 ± 2.1	13.2 ± 3.2	0.215	15.2 ± 3.8
First‐episode, yes/no	26/18	19/16	0.668	NA	25/3	14/2	1.000	NA
Duration of illness (mon)	18.8 ± 23.4	26.5 ± 32.9	0.233	NA	10.4 ± 12.7	18.3 ± 29.7	0.330	NA
PANSS score at baseline								
Total score	98.5 ± 20.0	90.2 ± 12.4	0.028	NA	85.9 ± 18.4	89.5 ± 12.7	0.488	NA
Positive score	24.6 ± 6.4	22.6 ± 7.1	0.184	NA	22.3 ± 6.3	22.1 ± 5.4	0.947	NA
Negative score	23.4 ± 9.5	22.9 ± 6.7	0.804	NA	18.9 ± 6.7	20.8 ± 9.2	0.440	NA
General psychopathology score	50.2 ± 9.2	44.7 ± 7.6	0.004	NA	44.7 ± 9.5	46.6 ± 4.5	0.378	NA
PANSS score at discharging								
Total score	67.3 ± 15.8	80.3 ± 11.1	<0.001	NA	54.4 ± 9.9	79.2 ± 9.8	<0.001	NA
Positive score	16.2 ± 4.5	19.6 ± 5.8	0.004	NA	13.0 ± 4.5	18.6 ± 4.2	<0.001	NA
Negative score	16.3 ± 6.1	20.7 ± 6.0	0.002	NA	11.9 ± 3.4	20.7 ± 6.8	<0.001	NA
General psychopathology score	34.8 ± 8.3	40.0 ± 6.0	0.002	NA	29.4 ± 5.1	39.9 ± 4.2	<0.001	NA
Stay in hospital (d)	20.3 ± 11.4	17.3 ± 7.9	0.181	NA	20.2 ± 8.6	14.8 ± 3.1	0.005	NA
Treatment without/with ECT	32/12	29/6	0.286	NA	17/11	12/4	0.336	NA
Treatment without/with rTMS	44/0	35/0	NA	NA	1/27	0/16	1.000	NA
Antipsychotic dose, mg/d^a^	11.8 ± 4.7	10.9 ± 4.9	0.420	NA	11.7 ± 4.0	9.4 ± 3.5	0.056	NA
Risperidone (%)	24 (55)	26 (74)	NA	NA	17 (61)	9 (56)	NA	NA
Olanzapine (%)	15 (34)	7 (20)	NA	NA	9 (32)	2 (13)	NA	NA
Haloperidol (%)	7 (16)	8 (23)	NA	NA	5 (18)	1 (6)	NA	NA
Ziprasidone (%)	7 (16)	1 (3)	NA	NA	1 (4)	0 (0)	NA	NA
Quetiapine (%)	4 (9)	6 (17)	NA	NA	1 (4)	0 (0)	NA	NA
Paliperidone (%)	3 (7)	3 (9)	NA	NA	5 (18)	3 (19)	NA	NA
Aripiprazole (%)	2 (5)	3 (9)	NA	NA	3 (11)	1 (6)	NA	NA
Chlorpromazine (%)	2 (5)	1 (3)	NA	NA	1 (4)	0 (0)	NA	NA
Perphenazine (%)	2 (5)	0 (0)	NA	NA	0 (0)	0 (0)	NA	NA
Amisulpride (%)	1 (2)	1 (3)	NA	NA	3 (11)	1 (6)	NA	NA
Sulpiride (%)	1 (2)	0 (0)	NA	NA	0 (0)	0 (0)	NA	NA
Clozapine	0 (0)	0 (0)	NA	NA	1 (4)	0 (0)	NA	NA
Changes in PANSS score, %	47 ± 14	16 ± 11	<0.001	NA	56 ± 14	17 ± 8	<0.001	NA
Changes in HAMD, %^b^	49 ± 19	35 ± 24	0.049	NA	31 ± 29	34 ± 16	0.732	NA
Changes in HAMA, %^b^	51 ± 20	23 ± 18	<0.001	NA	35 ± 18	21 ± 26	0.127	NA

ECT, electroconvulsive therapy; HAMA, Hamilton Anxiety Scale; HAMD, Hamilton Depression Scale; HCs, healthy controls; PANSS, Positive and Negative Syndrome Scale.

a Dose of current antipsychotic medication at time of MRI was converted to Defined Daily Dose (DDD) (Leucht et al., 2016).

b Data for HAMD/HAMA were available in 38 and 26 patients of principal dataset and replication dataset, respectively.

In order to validate the generalizability of our findings, we also enrolled a further 150 participants on another MRI scanner at a different time period as the replication dataset. This included 44 patients who met the criteria for schizophrenia spectrum disorder (schizophrenia, schizophreniform disorder, and brief psychotic disorder), diagnosed between April 2015 and December 2017 and 106 healthy controls. The exclusion criteria were also applied to this sample of patients. A subset of participants included in this study have been previously reported; 28 of the 79 patients in the principle dataset (Cui et al., 2016; Cui, Liu et al., 2017) and 23 of the 44 patients in the replication dataset (Cui et al., 2018). These articles deal with neural substrates of auditory verbal hallucinations and disease definition using functional connectivity, whereas in this manuscript we report on the predictive capacity of brain activity after early treatment for schizophrenia.

### 2.3 Image acquisition

For the principal dataset, images were collected on a 3.0 T Siemens Magnetom Trio Tim scanner. To acquire images of the independent replication dataset, a GE Discovery MR750 3.0 T scanner (similar sequences and specification) was used. High‐resolution T1‐weighted structural data and resting‐state BOLD data were obtained as performed previously (Supporting information Table S1). Further details about image acquisition are detailed in previous articles (Chang et al., 2015; Cui et al., 2015). Participants were instructed to relax but remain alert, and to keep their eyes closed and head still for the duration of their MRI scan. They were also asked to avoid lots of mental activity.

### 2.4 Data processing

Image processing was performed using Data Processing Assistant for Resting‐State fMRI Advanced Edition (DPARSFA) V4.3 within a toolbox for Data Processing & Analysis of Brain Imaging V2.3 (DPABI) (http://rfmri.org/dpabi). Data preprocessing and ALFF calculation were performed using previously published protocols (Cui et al., 2016). Briefly, we implemented realignment to correct the head motion (<3.0 mm translation and <3.0° rotation), resampled to 3 × 3 ×3 mm voxels, applied spatial smoothing with a Gaussian kernel (4 mm full width at half maximum) and band‐pass filtering (0.01–0.08 Hz), calculated ALFF and its standardization (ALFF value of each voxel divided by the global mean values), and performed Fisher *r*‐to‐*z* transformation of ALFF values. White matter signal and cerebrospinal fluid signal were selected as nuisance factors in nuisance covariates regression. We calculated the Jenkinson's mean frame‐wise displacement as a measure of the micro‐head motion of each subject, as we previously implemented (Cui, Chen et al., 2017), however, no difference was detected between responders and non‐responders (Supporting information Table S2). Peak Montreal Neurological Institute (MNI) coordinates with a radius of 6 mm were used for creating ROIs to extract ALFF values of each subject. ALFF_ratio_ refers to the ALFF value in the region where responders differed from non‐responders of each patient, divided by the mean ALFF value of the health controls, in the same region.

### 2.5 Statistical analysis

A two‐sample *t* test was performed for voxel‐based comparison of ALFF between responders and non‐responders in Statistical Parametric Mapping (SPM) 8 (https://www.fil.ion.ucl.ac.uk/spm/software/spm8/). A *p* value <0.001 (uncorrected) was considered as statistical significance for the whole brain analysis. The comparison of ALLF_ratio_ between responders and non‐responders was also performed using two‐sample *t* test. Regression of treatment type (presence/absence of ECT and atypical/atypical combined with typical antipsychotic), drug dose, and Jenkinson's mean frame‐wise displacement as covariates was also applied when performing the comparison. Afterward, these factors as covariates were included in the general linear model for ALFF_ratio_. ROC and Spearman correlation analyses were used to assess the predictive capacity and clinical relevance of regional brain function. The critical level was set at *p* < 0.05 for statistical significance.

### 2.6 Replication

In the replication dataset, the ALFF values were extracted for each subject using the ROI created, that is the left postcentral gyrus/inferior parietal lobule (*x* = −39, *y* = −42, *z* = 60; radius = 6 mm). After normalization by healthy controls, the ALFF_ratio_ was compared between responders and non‐responders, and then ROC and correlation analyses were performed.

## 3 RESULTS

### 3.1 Participant characteristics

Table 1 presents the demographic and clinical characteristics of the participants. Of the principal dataset, 44 and 35 patients were classified as responders (55.7%) and non‐responders (44.3%) to the treatment given during hospitalization which is consistent with previous reports (Sarpal et al., 2016). Except PANSS general psychopathology score, there was no statistically significant difference in other characteristics between responders and non‐responders at baseline. Participants who responded had a higher level of PANSS general psychopathology score (*p* = 0.004) prior to treatment than those who did not respond. The group of healthy controls used for normalization of ALFF included 87 subjects who had the same gender distribution (51 men and 36 women) as the patient group. The replication dataset included 28 responders (63.6%) and 16 non‐responders (36.4%), as well as 106 healthy controls. For responders, there was statistical significance in being younger (*p* = 0.014) and having a longer stay in hospital (*p* = 0.005) and a trend‐level significance in receiving more antipsychotic drugs (*p* = 0.056). Examining the predictive capacity of these clinical characteristics with difference is not feasible, because it is the case for either the principal dataset or the replication dataset.

### 3.2 ALFF difference in responders versus non‐responders

**Figure 2:**
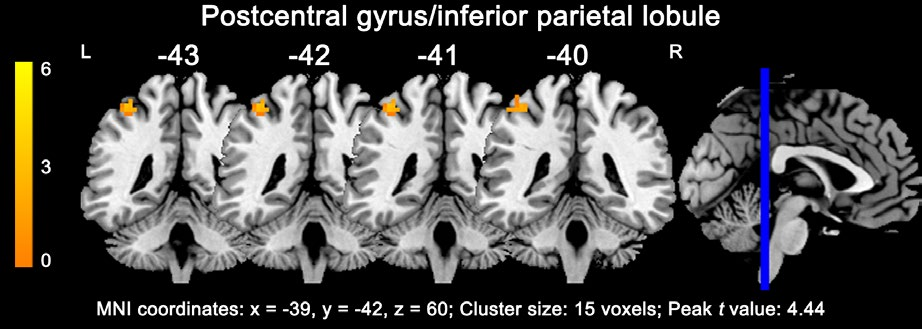
Amplitude of low‐frequency fluctuation (ALFF) difference in the left postcentral gyrus/inferior parietal lobule between responders and non‐responders. As compared with non‐responders, responders had a significantly increased ALFF in the left postcentral gyrus/inferior parietal lobule (*p* < 0.001, uncorrected)

**Figure 3:**
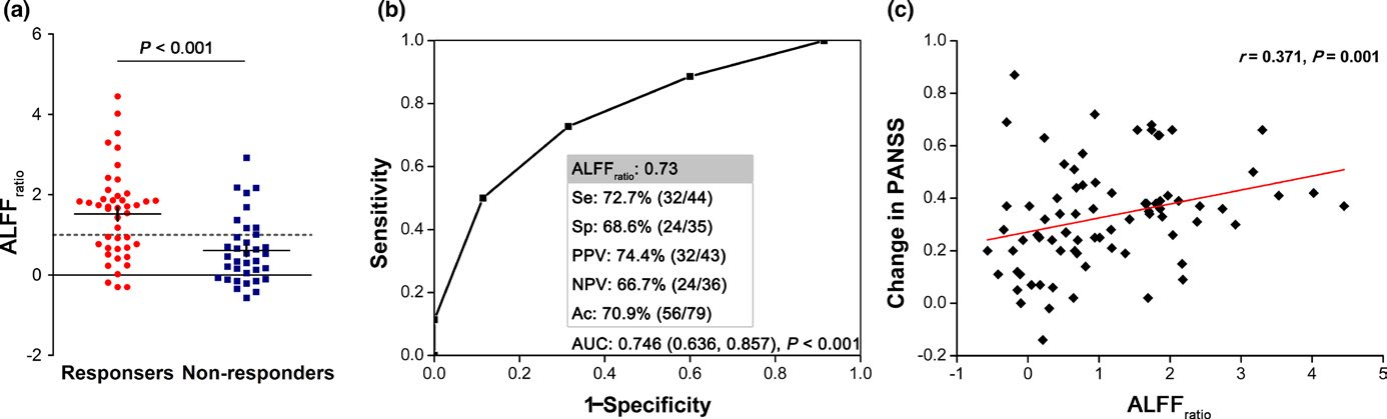
Predictive function of ALFF_ratio_ in the principal dataset. (a) Examining the ALFF_ratio_ showed a higher level in responders than non‐responders (*p* < 0.001). (b) The area under the ROC curve was 0.746 (*p* < 0.001; 95% CI, 0.636, 0.857). (c) In schizophrenia patients, ALFF_ratio_ was positively related to changes in PANSS (*r* = 0.371, *p* = 0.001)

Before treatment, responders exhibited only one cluster with more than 10 voxels as compared with non‐responders in the whole brain analysis, showing increased ALFF in the left postcentral gyrus and inferior parietal lobule (15 voxels; *x* = −39, *y* = −42, *z* = 60; peak *t* value = 4.44; Figure 2). Supporting information Table S3 summarizes the ALFF_ratio_ values in ROIs made of the regions where responders differed from non‐responders. Considering the application of this method clinically, we selected the left postcentral gyrus/inferior parietal lobule with a relatively large enough ROI size and statistical power to be used as a potential marker. Patients who subsequently responded to treatment had a higher ALFF_ratio_ at baseline compared to non‐responders (1.52 ± 1.11, 0.61 ± 0.82; *p* < 0.001, Figure 3a; *F* = 3.661, *p* = 0.006 in the general linear model).

### 3.3 ALFF_ratio_ correlates and predictive capacity of subsequent treatment response

The area under the ROC curve for ALFF_ratio_ was 0.746 (*p* < 0.001; 95% CI, 0.636, 0.857) to distinguish responders from non‐responders. Notably, when ALFF_ratio_ in the left postcentral gyrus/inferior parietal lobule was set at 0.73, sensitivity, specificity, and accuracy was 72.7%, 68.6%, and 70.9%, respectively (Figure 3b). After excluding patients treated with ECT, sensitivity and specificity was 71.9% and 72.4%, respectively (Supporting information Results). Additionally, we analyzed data focusing on positive symptom and length of stay; this did not strengthen the findings (Supporting information Results). There was a significant association between ALFF_ratio_ and changes in PANSS (*r* = 0.371, *p* = 0.001) and HAMA (*r* = 0.415, *p* = 0.010), as well as the natural log transformed illness duration (*r* = −0.368, *p* = 0.001) for patients (Figure 3c). No correlation was found between ALFF_ratio_ with the natural log transformed length of stay for the current hospitalization (*r* = 0.027, *p* = 0.815) and changes in HAMD (*r* = 0.274, *p* = 0.096).

### 3.4 Replication

**Figure 4:**
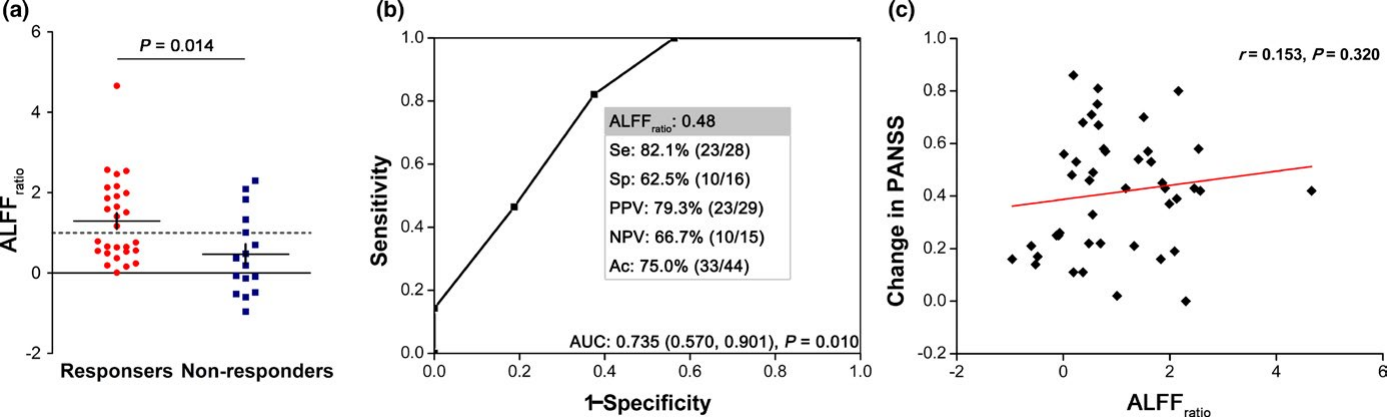
Replication results. (a) Confirming the findings above, responders had an increased level of ALFF_ratio_ (*p* = 0.014) relative to non‐responders in the independent replication dataset. (b) Similarly, the area under the ROC curve was 0.735 (*p* = 0.010; 95% CI: 0.570, 0.901). (c) The correlation between ALFF_ratio_ and change in PANSS did not remain significant (*r* = 0.153, *p* = 0.320)

Finally, we replicated the above findings in an independent dataset to test the association between clinical outcome and ALFF_ratio_ in the same area identified in the principal dataset (Figure 4). The ALFF_ratio_ in the left postcentral gyrus/inferior parietal lobule revealed a significant separation between responders and non‐responders (1.293 ± 1.037, 0.466 ± 1.001; *p* = 0.014). In the ROC analysis, similar results were observed for calculating the sensitivity (82.1%), specificity (62.5%), and accuracy (75.0%) for prediction, as well as area under ROC curve (0.735 [*p* = 0.010; 95% CI: 0.570, 0.901]), although the correlation did not remain significant between ALFF_ratio_ and change in PANSS (*r* = 0.153, *p* = 0.320), HAMA (*r* = −0.019, *p* = 0.928), or the natural log transformed illness duration (*r* = 0.108, *p* = 0.483). Confirming the results of principal dataset, no correlation was found between ALFF_ratio_ with the natural log transformed length of stay for the current hospitalization (*r* = −0.041, *p* = 0.794) and changes in HAMD (*r* = −0.032, *p* = 0.875).

## 4 DISCUSSION

This naturalistic study sought to predict early treatment response for the current hospitalization in a schizophrenia sample using regional brain function, which was validated in an independent sample. We identified ALFF in the left postcentral gyrus/inferior parietal lobule modified by a group of healthy controls with successful prediction of early treatment response. The principal findings indicate predictive capacity between patients who subsequently responded and did not respond, with clinically meaningful sensitivity and specificity. Meanwhile, significant association was found between ALFF_ratio_ and clinical characteristics in relation to future treatment response. Furthermore, we confirmed the generalizability of applying this measure for predicting treatment response in an independent sample of patients.

The suggestions from radiologists and psychiatrists to patients and their families regarding treatment efficacy, based on predictive/prognostic markers, are of remarkable clinical significance, especially as we move toward delivering precision medicine (https://allofus.nih.gov/). First, ALFF_ratio_ in the present study may assist in the treatment selection and personalization of treatment algorithms. Specifically, for patients who show no response within the first two weeks of early treatment, this may predict a failure in the future (Leucht, Busch, Kissling, & Kane, 2007; Lin et al., 2018; Murawiec & Boutros, 2012; Samara et al., 2015). In other words, patients classified as non‐responders at the end of the current hospitalization are unlikely to be responsive to conventional dopamine D2 antagonists, or to other similar antipsychotics that work by the same mechanism. Hence, additional therapies need to be considered and much more attention needs to be paid to ways of mitigating patient symptoms, such as transcranial magnetic stimulation (TMS), and psychological therapy. Secondly, for responders predicted at an early stage, enhancing the adherence to care through education and guidance for patients and their families by psychiatrists and radiologists according to ALFF_ratio_ could reduce rates of relapse.

Of particular interest, in our study, the high level of ALFF_ratio_ in the left postcentral gyrus/inferior parietal lobule at baseline indicates a better outcome after treatment during hospital stay. The speculative interpretation could include two points. From the perspective of neuropharmacology, in the clinical setting, dopamine receptor D2 blockade is the common mechanism of almost all antipsychotics. Treatment with clozapine and haloperidol up‐regulates D2 receptors in the postcentral gyrus (somatosensory cortex, areas 1, 2, and 3 of Brodmann) (Lidow & Goldman‐Rakic, 1994), suggesting this cortex as one of the brain regions affected by antipsychotic drugs. Initially, a relatively high level of regional activity might reflect its sensitivity to treatment in patients with schizophrenia. Furthermore, according to the structural and functional neuroimaging results, in a volumetric MRI study (Huang et al., 2015), a group of schizophrenia patients exhibit reduced gray matter volume in contrast to healthy controls in the left inferior parietal lobule (144 voxels; *x* = −43.5, *y* = −57, *z* = 49.5), a similar location detected by the current study. Gray matter density in the somatosensory and parietal cortices accurately predicts individual responses to repetitive TMS in patients with predominant negative schizophrenia (Koutsouleris et al., 2017). Meanwhile, alterations in the encoding force in the somatosensory domain of schizophrenia are observed in the postcentral gyrus (*x* = −38, *y* = −22, *z* = 56; *x* = −34, *y* = −57, *z* = 48) (Martinelli, Rigoli, & Shergill, 2017), showing approximately the same cerebral area used for response prediction in the responders at baseline. The postcentral gyrus/inferior parietal lobule serving as a marker makes sense at the neuroanatomical level.

Hospitalization is indeed required for a portion of schizophrenia patients, which functions as establishing a diagnosis, stabilizing medication, managing acute exacerbations and comorbid conditions, and maintaining safety of patients and the community. Predicting early response of the first/a single hospitalization seems to be considerably cost‐effective. Methodologically, measuring ALFF is not a complicated technique via the software available online for clinicians. Besides the convenience, normalization using healthy controls presents a full view of patients’ distribution across normal population, facilitating the feasibility to utilize ALFF_ratio_ to predict response before treatment at different sites. This step by step analysis could promote our findings transforming clinical practice, which is helpful for the generalizability of the potential tool.

Finally, we systematically reviewed clinical studies within the past five years to predict the outcome of patients with schizophrenia and/or other psychotic disorders (Supporting information Table S4) using MRI, widely including high‐resolution T1‐weighted imaging (Altamura et al., 2017; Dusi et al., 2017; Francis et al., 2018; Fung et al., 2014; Hutcheson et al., 2014; Molina et al., 2014; Morch‐Johnsen et al., 2015; Palaniyappan et al., 2013; Premkumar et al., 2015), BOLD‐fMRI (Hadley et al., 2014; Kraguljac, White, Hadley, Visscher et al., 2016; Kraguljac, White, Hadley, Hadley et al., 2016; Li, Jing et al., 2017; Nejad et al., 2013; Sarpal et al., 2016), diffusion tensor imaging (Crossley et al., 2017; Reis Marques et al., 2014), and arterial spin labeling (Stegmayer et al., 2017). In spite of relatively low predictive capacity (72.7%‐82.1% sensitivity and 62.5%‐68.6% specificity) compared with tests previously discovered (80% sensitivity and 75% specificity) (Sarpal et al., 2016), we propose this novel tool for early prediction as an alternative approach to contribute to the current potential markers. Future research needs to focus on integration and optimization of these tools in order to establish an accurate predictor for the clinical treatment and management of schizophrenia.

An obvious limitation is that the study patient population did not undergo identical treatments; their therapies involving antipsychotics were varied which therefore may introduce an effect on the results. This is a drawback of the naturalistic study design. It appears that the ALFF is predictive regardless of the type of therapy used by previous studies (Crossley et al., 2017; Palaniyappan et al., 2013; Reis Marques et al., 2014; Sarpal et al., 2016; Stegmayer et al., 2017). Notably, according to several recent findings, most neuroimaging studies of ECT in patients with major depressive disorder demonstrate treatment‐related increase of hippocampal volume (Oltedal et al., 2018; Redlich et al., 2016) and successful prediction for responsiveness using subgenual anterior cingulate volume (Redlich et al., 2016). However, a portion of the study group received ECT (and/or rTMS). Presumably, this intervention makes interpretation of the findings much more challenging. Furthermore, the characteristics of the patients with schizophrenia in this study, before treatment and prognosis, were varied. This led to unmatched ages in the replication dataset and an intersection of ALFF_ratio_ between responders and non‐responders. Although we focus on the predictive value of neuroimaging findings in our study, Supporting information Table S3 shows the difference with no statistical power (*q*_FDR correction_ = 0.158) in the left postcentral gyrus/inferior parietal lobule with a small cluster. Likewise, responders and non‐responders reveal quite a bit of overlapping in striatal connectivity (Sarpal et al., 2016) and global efficiency (Crossley et al., 2017), which indeed restricts the interpretability of the findings as predictors for schizophrenia.

## 5 CONCLUSIONS

Taken together, our results support the use of regional brain function as a potential tool for predicting treatment response during a single hospitalization in patients with schizophrenia, paving the way for the utilization of biomarkers in mental disorders. Additional research effort is vital for the standardization of psycho‐neuroimaging protocols which are required to achieve generalizability across independent sites, especially with regard to crosstalk and collaboration among radiologists, psychiatrists, and patients with psychosis.

## CONFLICT OF INTEREST

No conflict of interests.
