# Supplementary material for: Prediction of early response to overall treatment for schizophrenia: A functional magnetic resonance imaging study
Source: Brain Behav. 2019 Jan 30;9(2):e01211. doi: 10.1002/brb3.1211 (PMC6379641; doi:10.1002/brb3.1211)
Supplement: Supplementary file 1 [file BRB3-9-e01211-s001.docx]

**Supplementary Results**

We performed ROC analysis after excluding patients treated with ECT, suggesting a slight enhancement of specificity (specificity, from 68.6% to 72.4%; sensitivity, from 72.7% to 71.9%). Area under ROC curve for ALFF_ratio_ was 0.743 (*P* = 0.001; 95% CI, 0.615, 0.871) to distinguish responders from non-responders. When ALFF_ratio_ in the left postcentral gyrus/inferior parietal lobule was set at 0.9, sensitivity and specificity was 71.9% and 72.4%, respectively.

We then analyzed data focusing on positive symptom, which does not strengthen the findings. PANSS positive score percentage change = (PANSS endpoint positive score – PANSS baseline positive score) × 100 ÷ (PANSS baseline positive score – 7). Responder was defined as a 30% reduction. Patients who subsequently respond to treatment had a higher ALFF_ratio_ at baseline compared to non-responders (1.44 ± 1.12, 0.78 ± 0.95; *P* = 0.007). Area under ROC curve for ALFF_ratio_ was 0.683 (*P* = 0.005; 95% CI, 0.565, 0.801) to distinguish responders from non-responders. When ALFF_ratio_ in the left postcentral gyrus/inferior parietal lobule was set at 0.8, sensitivity, specificity, and accuracy was 65.0% and 61.5%, respectively. There was a significant association between ALFF_ratio_ and changes in PANSS positive score (*r* = 0.284, *P* = 0.011) for patients.

Furthermore, there was no significant association between changes in PANSS and the natural log transformed length of stay for the current hospitalization for patients (*r* = 0.216, *P* = 0.056). ROC analysis showed an area under curve of 0.609 (*P* = 0.097; 95% CI: 0.480, 0.738) to distinguish responder from non-responders.

**Table 1.** Detailed Scanning Parameters

|  | Siemens scanner | |  | GE scanner | |
| --- | --- | --- | --- | --- | --- |
|  | T1 | BOLD |  | T1 | BOLD |
| Repetition time (ms) | 2530 | 2000 |  | 8.2 | 2000 |
| Echo time (ms) | 3.5 | 30 |  | 3.2 | 30 |
| Flip angle (°) | 7 | 90 |  | 12 | 90 |
| Field of view (mm^2^) | 256 × 256 | 220 × 220 |  | 256 × 256 | 240 × 240 |
| Matrix | 256 × 256 | 64 × 64 |  | 256 × 256 | 64 × 64 |
| Slice thickness (mm) | 1 | 4 |  | 1 | 3.5 |
| Section gap (mm) | 0 | 0.6 |  | 0 | 0 |
| Number of slices | 192 | 33 |  | 196 | 45 |

**Table 2.** Comparison of the Jenkinson’s mean frame-wise displacement Between Responders and Non-responders

| Dataset | Responders | Non-responders | *P* value |
| --- | --- | --- | --- |
| Principal dataset | 0.090 ± 0.060 | 0.095 ± 0.062 | 0.686 |
| Replication dataset | 0.079 ± 0.069 | 0.051 ± 0.027 | 0.122 |

**Table 3.** Comparison of ALFF/ALFF_ratio_ Between Responders and Non-responders

|  | Peak MNI coordinate | | |  |  |  |  | ALFF_ratio_ extracted using ROIs | |  |
| --- | --- | --- | --- | --- | --- | --- | --- | --- | --- | --- |
|  | x | y | z | Cluster size | *T* value | *P* value | *q*_FDR correction_ | Responders | Non-responders | *P* value |
| Left postcentral gyrus/inferior parietal lobule | -39 | -42 | 60 | 15 | 4.44 | < .001 | .158 | 1.52 (1.11) | 0.61 (0.82) | < .001 |
| Left insula | -42 | -6 | 0 | 2 | 3.83 | < .001 | .534 | 0.01 (7.13) | -3.83 (3.40) | .002 |
| Right medial superior frontal gyrus | 6 | 36 | 57 | 1 | 3.37 | .001 | .988 | 1.52 (1.21) | 0.82 (0.97) | .007 |
| Right postcentral gyrus | 60 | -12 | 42 | 1 | 3.28 | .001 | .988 | 0.72 (2.03) | -0.21 (1.57) | .029 |

MNI, Montreal Neurological Institute; ROIs, regions of interest.

**Table 4.** Studies on Prediction of Treatment Response in Schizophrenia and/or Psychosis using Baseline MRI^*^

| **Modalities** | **References** | **Subjects** | **Treatments** | **Intervals** | **Questionnaires** | **Summary of results** |
| --- | --- | --- | --- | --- | --- | --- |
| **HR T1WI** | Francis MM, Hummer TA, Vohs JL *et al*. ***Brain Imaging Behav***, 2018 May 31. doi: 10.1007/s11682-018-9902-4. | 20 early phase psychosis | Active or sham rTMS, and antipsychotics | 2 weeks | Brief Assessment of  Cognition | Greater improvement in cognitive function: thicker left caudal middle frontal cortical thickness |
|  | Altamura AC, Delvecchio G, Paletta S *et al*. ***Psychiatry Res***, 2017; 261:80-84. | 7 acute psychosis | Paliperidone palmitate long acting injectable | 24 weeks | BPRS | Better improvement: higher total gray matter volume |
|  | Dusi N, Bellani M, Perlini C *et al*. ***Schizophr Res***, 2017; 179:104-111. | 70 schizophrenia; 76 healthy controls | Antipsychotics | 3 years | BPRS; WHO-DAS-2 | Poor outcome: decreased right dorsolateral prefrontal cortex white matter |
|  | Mørch-Johnsen L, Nesvåg R, Faerden A *et al*. ***Schizophr Res***, 2015; 164(1-3):59-64. | 70 first-episode psychosis | Antipsychotics | 1 year | Clinical version of Apathy Evaluation Scale | Patients with persistent apathy: thinner left orbitofrontal cortex and left anterior cingulate cortex |
|  | Premkumar P, Fannon D, Sapara A *et al*. ***Psychiatry Res***, 2015; 231(3):298-307. | 25 schizophrenia or schizoaffective disorder; 25 healthy controls | cognitive behavioural therapy for psychosis | 6-8 months | PANSS | Improvement in symptoms: positively correlated with OFC gray matter volume and rightward OFC asymmetry |
|  | Molina V, Taboada D, Aragüés M *et al*. ***Schizophr Res***, 2014; 158(1-3):223-9. | 31 first-episode schizophrenia | Risperidone or clozapine | 2 years | PANSS | Improvement in symptoms: negatively correlated with cortical thickness in the right prefrontal cortex (pars orbitalis) |
|  | Fung G, Cheung C, Chen E *et al*. ***Neuropsychobiology***, 2014; 69(4):243-8. | 39 schizophrenia | Antipsychotics | 1 year | Global Assessment of Functioning | Early remission: positively correlated with bilateral lentiform and striatal volumes for females |
|  | Hutcheson NL, Clark DG, Bolding MS *et al*. ***Psychiatry Res***, 2014; 221(1):6-12. | 23 schizophrenia | Risperidone | 6 weeks | BPRS | Improvements in symptoms: positively correlated with volume of the bilateral caudate |
|  | Palaniyappan L, Marques TR, Taylor H *et al*. ***JAMA Psychiatry***, 2013; 70(10):1031-40. | 80 first-episode psychosis; 46 healthy controls | Antipsychotics | 12 weeks | PANSS | Non-responders: hypogyria at bilateral insular, left frontal, and right temporal regions |
|  |  |  |  |  |  |  |
| **BOLD** | Shafritz KM, Ikuta T, Greene A *et al*. ***Brain Imaging Behav***, 2018 May 9. doi: 10.1007/s11682-018-9876-2. | 33 first-episode psychosis; 33 healthy controls | Risperidone (1-6 mg/d) or aripiprazole (5-30 mg/d) | 12 weeks | BPRS-A; CGI | Responders: greater activation in ACC for a simple response conflict task |
|  | Li P, Jing RX, Zhao RJ *et al*. ***NPJ Schizophr***, 2017; 3(1):21. | 34 schizophrenia; 34 healthy controls | antipsychotics only or a combination of antipsychotics and ECT | 6 weeks | PANSS | Better treatment outcome: lower classification scores of intrinsic connectivity networks |
|  | Kraguljac NV, White DM, Hadley N *et al*. ***Schizophr Bull***, 2016 Jul;42(4):1046-55. | 34 schizophrenia or schizoaffective disorder; 34 healthy controls | risperidone | 6 weeks | BPRS | Change in symptoms: correlated with hippocampal connectivity |
|  | Sarpal DK, Argyelan M, Robinson DG *et al*. ***Am J Psychiatry***, 2016; 173(1):69-77. | 41 first-episode schizophrenia spectrum disorder; 40 schizophrenia spectrum disorder and bipolar I disorder; 41 healthy controls | Risperidone (1-6 mg/d) or aripiprazole (5-30 mg/d) | 12 weeks | BPRS-A; CGI | Responders: Lower striatal functional connectivity |
|  | Kraguljac NV, White DM, Hadley JA *et al*. ***Neuroimage Clin***, 2016; 10:146-58. | 34 schizophrenia; 34 healthy controls | Risperidone | 6 weeks | BPRS | Change in symptoms: positively correlated with dorsal attention network |
|  | Hadley JA, Nenert R, Kraguljac NV *et al*. ***Neuropsychopharmacology***, 2014; 39(4):1020-30. | 21 schizophrenia; 21 healthy controls | Risperidone | 6 weeks | BPRS | Change in symptoms: positively correlated ventral tegmental area/midbrain connectivity strength to dorsal anterior cingulate cortex |
|  | Nejad AB, Madsen KH, Ebdrup BH *et al*. ***Int J Neuropsychopharmacol***, 2013; 16(6):1195-204. | 14 schizophrenia | Quetiapine | 7 months | PANSS | Negative symptom improvers: Classified accurately by bilateral frontoparietal and default mode networks |
|  |  |  |  |  |  |  |
| **DTI** | Crossley NA, Marques TR, Taylor H *et al*. ***Brain***, 2017; 140(2):487-496. | 76 first-episode psychosis; 74 healthy controls | Antipsychotics | 12 weeks | PANSS | Responders: higher global efficiency in the structural connectomes |
|  | Reis Marques T, Taylor H, Chaddock C *et al*. ***Brain***, 2014; 137(Pt 1):172-82. | 63 first-episode psychosis; 52 healthy controls | Antipsychotics | 12 weeks | PANSS | Non-responders: lower fractional anisotropy, mainly in the uncinate, cingulum and corpus callosum |
|  |  |  |  |  |  |  |
| **ASL** | Stegmayer K, Stettler M, Strik W *et al*. ***Acta Psychiatr Scand***, 2017; 136(5):506-516. | 47 schizophrenia spectrum disorder; 30 healthy controls | Antipsychotics | 6 months | Scale for the assessment of thought, language, and communication | Social functioning: positively correlated with perfusion within the left supramarginal gyrus |

^*^Search terms: (“schizophrenia”[Mesh] OR “psychosis”[All Fields] OR “schizo*”[All Fields]) AND (“response”[All Fields] OR “outcome”[All Fields]) AND (“antipsychotic”[All Fields] OR “treatment”[All Fields] OR “therapy”[All Fields]) AND “magnetic resonance imaging”[All Fields] AND (“baseline”[All Fields] OR “longitudinal”[All Fields])

ACC, anterior cingulate cortex; ASL, arterial spin labeling; BOLD, blood oxygen level-dependent; BPRS-A, Brief Psychiatric Rating Scale-Anchored Version; CGI, Clinical Global Impressions Scale; DTI, diffusion tensor imaging; ECT, electroconvulsive therapy; HR T1W, high-resolution T1-weighted imaging; OFC, orbitofrontal cortex; PANSS, Positive and Negative Symptom Scale; rTMS, repetitive transcranial magnetic stimulation; WHO-DAS-2, World Health Organization Disability Assessment Scale 2
